# Targeting the tomato fruit epidermis using the *nsLTP2* promoter: a molecular tool to study the composition, architecture and properties of the cuticle

**DOI:** 10.1186/s13007-026-01559-w

**Published:** 2026-06-20

**Authors:** Marie Alonso, Ella Paulsen, Nathalie Geneix, Sandra Pedemay, Angelina D’Orlando, Didier Marion, Marc Lahaye, Benedicte Bakan, Christophe Rothan, Johann Petit

**Affiliations:** 1 Univ. Bordeaux, INRAE, UMR BFP, Villenave d’Ornon, Bordeaux, 33140 France; 2 Unité Biopolymères, Interactions, Assemblages, INRAE, BP71627, Nantes Cedex 3, 44316 France; 3 PROBE Research Infrastructure, Biopolymers Interactions, Structural Biology (BIBS) Facility, BP71627, Nantes Cedex 3, 44316 France

**Keywords:** Non-specific Lipid Transfer Protein, Promoter, Fruit cuticle, *Solanum lycopersicum*, Epidermis, CRISPR-TSKO, RAMAN microspectroscopy

## Abstract

**Background:**

The cuticle is a hydrophobic polymeric assembly covering the surface of the aerial parts of plants. In the tomato fruit (*Solanum lycopersicum*), the cuticle is involved in many agronomically important traits, such as fruit brightness, resistance to cracking or shelf life. The development of a molecular tool targeting cuticle-associated genes in tomato fruit epidermis could considerably advance our understanding of the relationship between the structure of the cuticle and its functional properties.

**Results:**

According to its expression profile, the *non-specific Lipid Transfer Protein 2* promoter (*pronsLTP*) was selected and its application as a molecular tool for targeting modifications in the epidermis of tomato fruit was validated. Indeed, fluorescence analysis confirmed that *pronsLTP* activity is associated with the fruit epidermis and coincides with cuticle accumulation. In addition, edition using a CRISPR tissue-specific knockout approach (CRISPR-TSKO) of the carotenoid *SlPSY1* gene via *pronsLTP* showed a high mutation frequency only in the fruit exocarp and mesocarp. Furthermore, overexpression of the flavonoid regulatory gene *SlMYB75* under the control of *pronsLTP* resulted in targeted accumulation of anthocyanins in the exocarp of growing fruit, associated with a change in cuticle composition and permeability.

**Conclusions:**

Altogether, our results provide a new molecular tool essential for delineating the functional role of cuticle components and opens new perspectives for the targeted engineering of cuticle-related traits to improve fruit quality and agronomical properties.

**Supplementary Information:**

The online version contains supplementary material available at https://doi.org/10.1186/s13007-026-01559-w.

## Background

One of the primary objectives for fruit growers is to produce high-quality fruit that can withstand transportation and storage. The fruit surface at the interface between the fruit and its environment, acts as a protective barrier against pests, pathogens and water loss. It therefore plays a crucial role in determining the shelf life of fruit and affecting its appearance (color, glossiness, regularity, cracking…) and nutritional value [1]. In tomato, the fruit exocarp (also called peel or skin) is made up of an epidermal cell layer covered with the cuticle, and of several layers of sub-epidermal cells whose function is to generate mesocarp cells in the growing fruit [2–5]. The mesocarp underneath is a fleshy and heterogeneous tissue consisting of large polyploid cells which accumulate water, soluble sugars and organic acids [2, 4]. The last cell layer of the pericarp facing the locular tissue is the endocarp. It consists of a single inner epidermal cell layer topped by the inner cuticle, both of which share common features with the outer epidermis and outer cuticle [6].

In addition to being a fleshy fruit model for which a large collection of genetic and genomic resources and tools is available [7], tomato recently emerged as a model for cuticle studies. Its thick, astomatous cuticle is easy to collect for study using a wide range of chemical and physical technologies [1, 8–12]. In addition, the availability of tomato natural and artificially-induced cuticle mutants and transgenic lines [13] has enabled the discovery of new cuticle-related functions and mechanisms [9, 14, 15].

The fruit cuticle is covered by an epicuticular film of waxes, which are also embedded within the cutin layer. Waxes are a complex mixture of derivatives of very-long-chain fatty acids (VLCFA), mainly alkanes and alcohols, and also include various secondary metabolites, such as amyrins, flavonoids and sterols [16]. The main component of the cuticle is cutin [17], which forms a continuum with cell wall (CW) polysaccharides of epidermal cells [18, 19]. Cutin is mainly constituted by a polyester of long chain hydroxy and epoxy fatty acids, fatty alcohols, and by varying amounts of phenolic compounds (e.g., p-coumaric acid and flavonoids) [11]. Like waxes, with which it shares common precursors, cutin precursors are synthesized in epidermal cells and finally exported to CW through ABC transporters [14]. In the CW, cutin precursors are transesterified by cutin synthases into a network of linear and branched cutin polymers [9, 17, 19]. A growing body of evidence suggests a role for non-specific Lipid-Transfer Proteins (nsLTP) in the extracellular trafficking of the lipid monomers required for cutin assembly in the cell wall [20].

Fruit skin properties largely depend on the composition and structure of the cuticle [8, 11]. Lipid polyesters interact with non-lipid components including cuticle-embedded polysaccharides (CEP) and phenolics to determine cuticle architecture and properties [11, 21, 22]. CEP are diverse (cellulose, hemicelluloses, pectins) and exhibit specific features such as a high degree of pectin esterification (methylation and acetylation) and a high cellulose crystallinity [15, 18]. Furthermore, it has been shown that the growing fruit cuticle undergoes compositional and macromolecular rearrangements such as the specific spatiotemporal accumulation of phenolic compounds (p-coumaric acid and flavonoids) and the dynamic remodeling of CEP [21, 22]. These multiple chemical remodelling have been associated to nanomechanical properties of the tomato cuticle [23].

Altogether, these findings suggest that properties and function of cuticles result from the assembly of cuticular lipids, cell wall polysaccharides and phenolics during fruit development. The individual impact of these different components on the architecture and properties of the cuticle is currently a major unanswered question. Much progress to decipher the function of cuticular lipid compounds has been made in recent decades thanks to cutin-deficient mutants targeting lipid genes whose expression is specific to epidermal cells [6, 24]. For CW compounds and polyphenols that fulfil important roles, expression occurs throughout entire tissues and organs [24]. It is therefore necessary to target them to the cuticle in order to study them accurately.

In this study, our aim is to provide a molecular tool to study the composition, the architecture and the properties of the cuticle using a promoter that targets the outermost parts of the fruit. Cuticular material is mainly deposited into the outer epidermal cell wall at early stages of fruit development [22, 25], while sub-epidermal cells may also synthesize smaller amounts of cuticular components [22, 26]. Therefore, this promoter should meet the following requirements: (i) be preferentially expressed in the fruit and in the fruit epidermis to avoid pleiotropic effects; (ii) target the early stage of fruit development, i.e. before 15–20 Days Post Anthesis (DPA), when the expression of cutin gene is at its peak [3]; (iii) be sufficiently potent to efficiently drive transgene expression in the fruit epidermis and induce changes in cuticle composition and properties.

To develop this molecular tool, we selected the promoter of a cuticle-related gene, the *non-specific Lipid Transfer Protein* (*nsLTP2*) gene and validated its expression pattern in the epidermal layers of the fruit. To demonstrate that we can target the modification of the fruit cuticle through the *nsLTP2* promoter (*pronsLTP*), we designed a proof-of-concept experiment using CRISPR-TSKO and overexpression approaches, targeting the *PHYTOENE SYNTHASE 1* (*PSY1) and* the *SlMYB75* transcription factor, two genes associated with the synthesis of fruit pigments. The effect of targeted overexpression of *SlMYB75* via *pronsLTP* on the cuticle composition and properties was investigated. Our results validate that *pronsLTP* is well suited for overexpressing target genes in the fruit epidermis, thus facilitating the investigation of the role of lipids, polysaccharides and phenolics to cuticle architecture and properties.

## Results

### Gene expression analysis reveals that the *non-specific Lipid Transfer Protein 2* (*nsLTP2*) promoter is a promising candidate for targeted modification of the cuticle

**Fig. 1:**
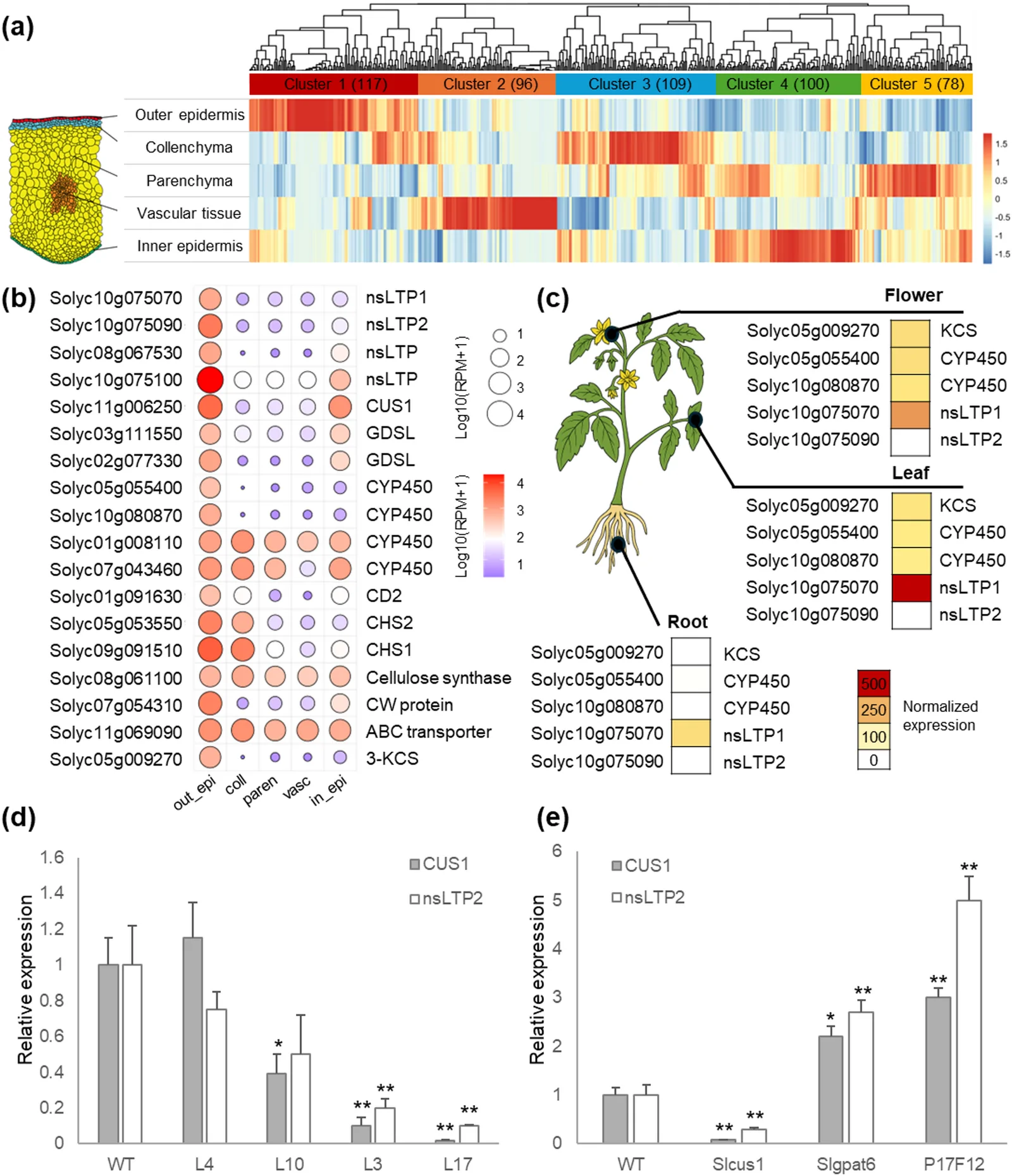
Tissue-specific expression pattern of cuticle-related genes in tomato fruit and plant organs. **a** Hierarchical clustering analysis of expressed genes in five tomato fruit tissues (outer epidermis, collenchyma, parenchyma, vascular tissue and inner epidermis). These genes were grouped into five cluster according to their expression profiles. Schematic representation of the five tissues was extracted from [24]. **b** Dot plot representing expression level (log10(RPM + 1)) of selected cuticle-related genes in the five tissues. out_epi, outer epidermis; coll, collenchyma; paren, parenchyma; vasc, vascular tissue; in_epi, inner epidermis. **c** Schematic representation of the normalized expression (extracted from [27]) of five candidate genes in three plant organs (flower, leaf and root). **d** *nsLTP2* expression in *cus1* RNAi transgenic lines L4, L10, L3 and L17; **e** *nsLTP2* expression in *cuticle-deficient* EMS mutants *cus1*, *gpat6*, and *cuticle-enriched* EMS mutant P17F12. Values are mean ± SD (*n* = 3). *, *P* < 0.05; **, *P* < 0.01 (Student’s t test)

To explore candidate promoters as molecular tools for targeted modification of the tomato fruit cuticle, we used tomato gene expression data previously obtained by laser microdissection and RNA-seq analysis of fruit tissues and cell-types throughout fruit development [24]. In line with previous cuticle studies [6, 12], we focused our gene expression analysis at 10 days post-anthesis (DPA) fruit stage, which corresponds to the peak of the increase in fruit volume and leads to the maximum rate of cuticle synthesis, which lasts until 20 DPA. To identify groups of genes preferentially expressed in the cuticle at fruit level, we first performed hierarchical clustering of the 500 most variable genes in five tissues: outer epidermis (first layer of epidermal cells, referring to the cuticle), collenchyma (sub-epidermal cells), parenchyma (mesocarp cells), vascular tissue and inner epidermis (Fig. 1A and Table S1). We identified five clusters, each representing a group of genes with high expression in one of the five fruit tissues (Fig. 1A and Table S1). Cluster 1 (117 genes) is associated with the most expressed genes in the outer epidermis, cluster 2 (96 genes) with vascular tissue, cluster 3 (109 genes) with collenchyma, cluster 4 (100 genes) with the inner epidermis and cluster 5 (78 genes) with parenchyma. We next investigated the expression of genes associated with cuticle biosynthesis and regulation in cluster 1 and we have selected 18 genes, including those encoding *non-specific Lipid Transfer Proteins* (*nsLTP*), lipases/esterases containing the *GDSL-motif*, *chalcone synthase* (*CHS*), *3-ketoacyl-CoA synthase* (*KCS*), proteins of the *cytochrome P450 family* (*CYP450*) (Fig. 1B and Table S2). Among these genes, *nsLTP1* (Solyc10g075070), *nsLTP2* (Solyc10g075090), *CYP450* (Solyc05g055400 and Solyc10g080870) and *KCS* (Solyc05g009270) are preferentially expressed in the outer epidermis while others, like *nsLTPs* (Solyc08g067530 and Solyc10g075100), *Cutin deficient 2 (CD2)*, *CHS* and *GDSL* genes, share a high level of expression (> 50 Reads Per million Mapped reads) with inner epidermal and/or collenchyma tissues (Fig. 1B and Table S2).

To ensure tissue specificity and avoid pleiotropic effects during genetic modifications, we used the expression data available on TomExpress, an RNA-seq data platform [27], to examine the expression of these five genes in three other plant organs: root, leaf and flower (Fig. 1C and Table S3). No expression of these genes in the root was found, with the exception of *nsLTP1*, which was expressed in all three organs, with very high expression in the leaf (Fig. 1C and Table S3). In the leaf and flower, gene expression analysis revealed that the *KCS* and *CYP450* genes were expressed in these organs, while *nsLTP2* showed no detectable expression (Fig. 1C and Table S3).

To further explore the link of *nsLTP2* with cutin deposition, the major component of the cuticle, we next analyzed its expression in *cutin-deficient* and *cutin-enriched* tomato mutants [9, 13, 25, 28]. In the *cus1* RNAi-silenced Wva106 cherry tomato transgenic lines, we observed the same pattern in *CUS1* and *nsLTP2* transcript abundance (Fig. 1D). We found the same trend between the abundance of *nsLTP* and *CUS1* transcripts in the Micro-Tom *cutin-deficient Slcus1* and *Slgpat6* and *cutin-enriched* P23F12 mutants (Fig. 1E and S1). These results suggest a complex yet unexplored relationship between *nsLTP2*, *CUS1* expression and cutin deposition. Taking together, these analyses strengthen the potential suitability of the *nsLTP2* promoter to drive the possible editing and overexpression of target genes in the fruit epidermis. This tool should enable the exploration of the contribution of cutin-embedded polysaccharides, phenolics and flavonoids to cuticle architecture and properties.

### The *nsLTP2* promoter drives *GFP* expression in fruit epidermis

**Fig. 2:**
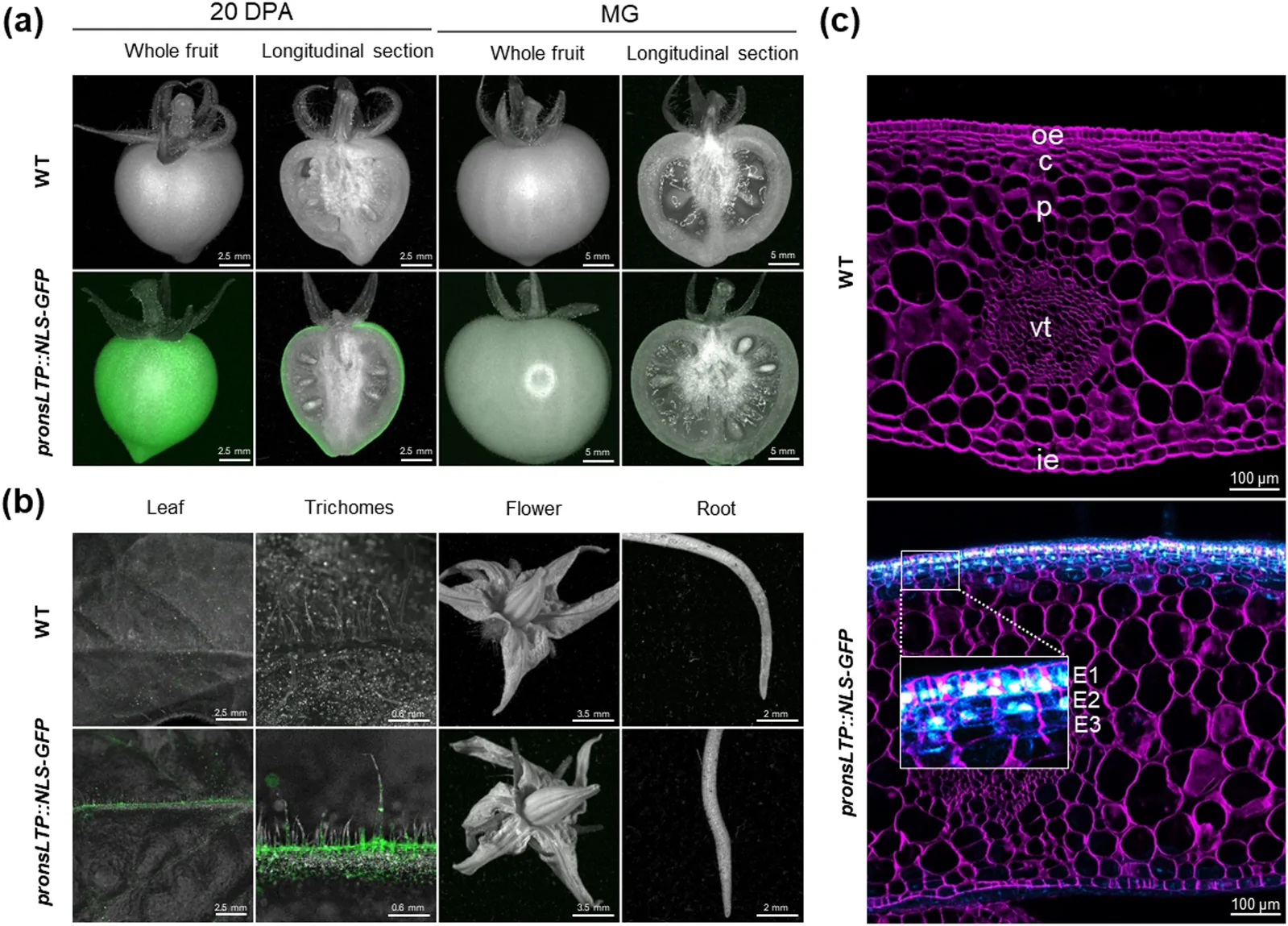
*pronsLTP*-driven nuclear GFP expression in tomato fruit tissues and plant organs. **a** Macroscopic detection of GFP fluorescence of *pronsLTP::NLS-GFP* fruit. Overlays of black-and-white and GFP images are shown for representative 20 DPA and MG fruits of *pronsLTP::NLS-GFP* and WT. DPA, Days Post Anthesis; MG, Mature Green. **b** Macroscopic detection of GFP fluorescence in leaves, trichomes, flowers and roots of *pronsLTP::NLS-GFP* and WT. Overlays of black-and-white and GFP images. **c** Confocal microscopy detection of GFP fluorescence (in blue) after calcofluor staining (in purple) of cross-sections of 5 DPA fruit pericarp of WT and *pronsLTP::NLS-GFP*. Inset, magnified image of exocarp showing localization of GFP in nucleus from E1, E2 and E3 cell layers of outer epidermis. oe, outer epidermis; c, collenchyma; p, parenchyma; vt, vascular tissue; i.e., inner epidermis; E1, outer epidermis, layer 1; E2, sub epidermis, layer 2; E3, sub epidermis, layer 3

In order to study and validate the localization of the *nsLTP2* promoter (*pronsLTP*) activity in vegetative and reproductive organs, we used the Green Fluorescent Protein (GFP) tagged with the nuclear localization signal (NLS) as reporter gene (Fig. 2). Observation of GFP labelling showed no signal in wild-type (WT), as expected, while the *pronsLTP::NLS::GFP* lines showed an intense bright green signal on the fruit surface until 20 DPA, which then decreased at the Mature Green (MG) stage, before the onset of ripening (Fig. 2A). Examination of the 20 DPA fruit cross-section showed that the green fluorescence was clearly restricted to the fruit skin (Fig. 2A). Although a signal was detected in leaf trichomes; we observed that none of the leaf epidermal cells showed any signal (Fig. 2B). In addition, and consistent with the distribution of *nsLTP2* transcripts in the plant (TomExpress) (Table S3), no *pronsLTP* activity was detected in other plant organs, e.g. flower and root (Fig. 2B). Accordingly, no pleiotropic effect should be expected when using *pronsLTP*.

To further analyze the spatial distribution of GFP in the fruit tissues of *pronsLTP::NLS-GFP*, we performed confocal analysis of 5 DPA fruit cross-sections stained with calcofluor to visualize cell walls (Fig. 2C). Intense GFP signal was observed in all cells of the outer epidermis (E1 cell layer). A decreasing signal was observed in the nuclei of sub-epidermal cells of the E2 layer as well as some sub-epidermal cells of the E3 layer (Fig. 2C). A weak fluorescence was also seen in cells of the inner epidermis, while no fluorescence was observed in the enlarging cells of the mesocarp, in agreement with expression analysis (Tables S1 and S2). These results indicate that *pronsLTP* activity in tomato fruit is restricted to the first three layers of the epidermis, with the strongest activity localized in the outer epidermis E1 layer. This suggests that the *nsLTP2* promoter is well-suited as a molecular tool to target the modification of the tomato fruit epidermis.

In silico analysis of the *nsLTP2* promoter identified several cis-regulatory elements associated with epidermal differentiation, light responsiveness and MYB-related regulation, which is consistent with the expression observed in fruit epidermal tissues and trichomes (Table S5). Taken together, these results indicate that *pronsLTP* is well suitable for efficient mutagenesis of cuticle-related genes within tomato fruit and limit pleiotropic effects.

### The *nsLTP2 promoter* drives gene editing of the carotenoid *SlPSY1* gene in the fruit epidermis

In a first attempt, we sought to assess whether *pronsLTP* could be used as a CRISPR-TSKO (tissue-specific knockout) approach. We targeted the *PHYTOENE SYNTHASE 1* (*SlPSY1*), a known gene involved in carotenoid biosynthesis [29], to provide a visual proof-of-concept that *pronsLTP* efficiently induce mutations in the cuticle of tomato fruit. *Cas9* expression was driven by *pronsLTP* with a single guide RNA (sgRNA) specific to the *SlPSY1* coding sequence. To evaluate the tissue specificity of the *pronsLTP*-driven *Cas9* activity, we sequenced leaves and two different fruit parts: exocarp and mesocarp.

**Fig. 3:**
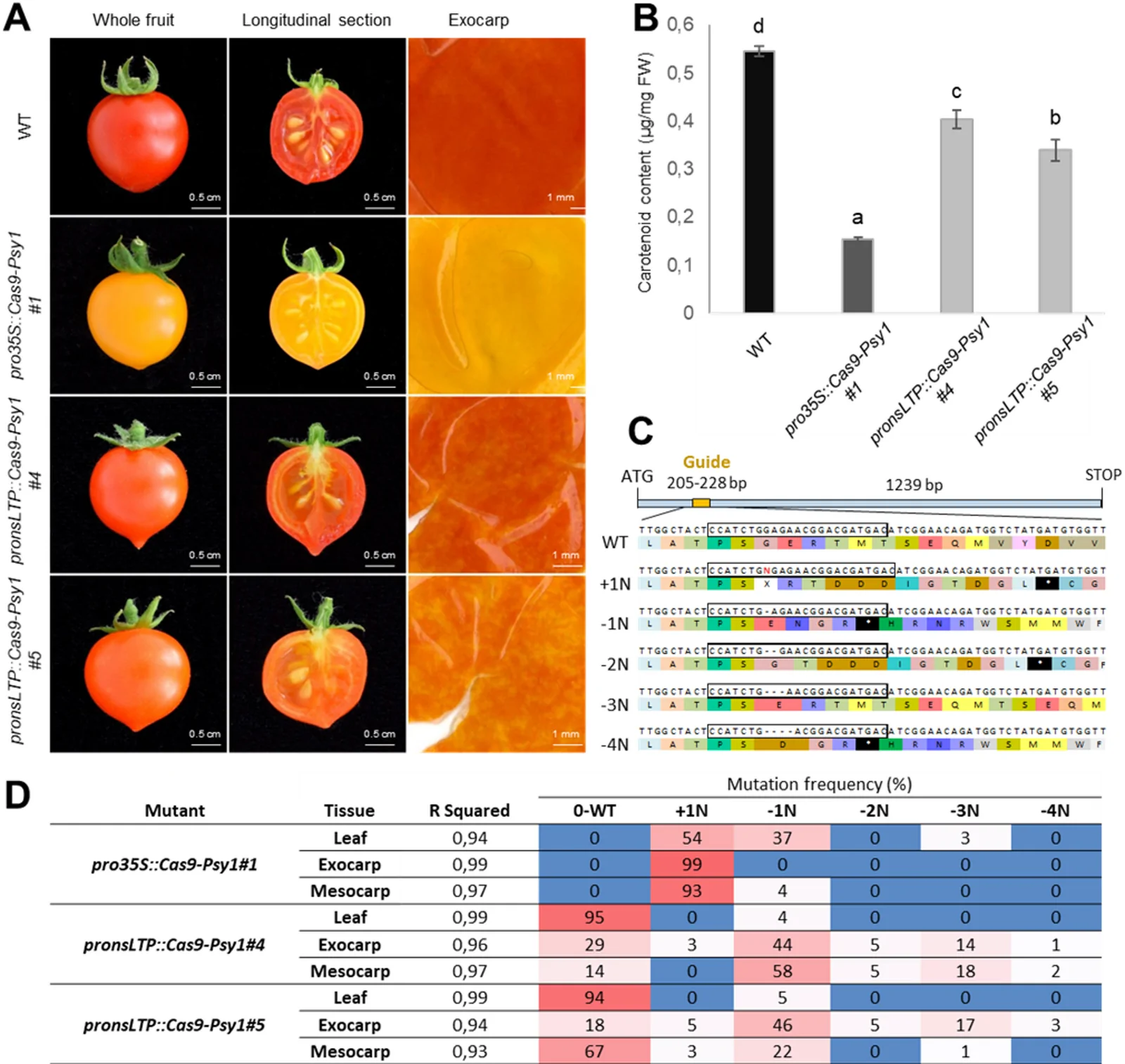
CRISPR tissue-specific knockout (CRISPR-TSKO) of phytoene synthase *SlPSY1* gene using *pronsLTP*. **a** Macroscopic observation of fruit color at RR stage, on whole fruit (left panel), longitudinal section (middle panel) and white light scan of a peeled exocarp (left panel), from WT, *pro35S::Cas9-Psy1#1*, *pronsLTP::Cas9-Psy1#4* and *pronsLTP::Cas9-Psy1#5* T1 plants. **b** Carotenoid content of RR fruit pericarp of WT, *pro35S::Cas9-Psy1#1*, *pronsLTP::Cas9-Psy1#4* and *pronsLTP::Cas9-Psy1#5* T1 plants. Values are mean ± SD (*n* = 3). Lower-case letters indicate significant differences (*P* < 0.05; Kruskal-Wallis test with Tukey post-hoc test). **c** Position of the guide (box) for *SlPSY1* targeting by the CRISPR/Cas9 system. The various mutations detected in *pro35S::Cas9-Psy1* and *pronsLTP::Cas9-Psy1* mutants are indicated below the WT sequence. CRISPR/Cas9-induced frameshift insertions and deletions led to truncated SlPSY1 proteins. N, nucleotide; * indicates a stop codon. **d** Frequency and type of mutations observed in leaf and fruit tissues (exocarp, mesocarp plus inner epidermis) of *pro35S::Cas9-Psy1#1*, *pronsLTP::Cas9-Psy1#4* and *pronsLTP::Cas9-Psy1#5* T1 plants

As expected, gene editing of *SlPSY1* under the control of the 2 × 35 S promoter (*pro35S::Cas9-Psy1*) produces yellow-colored fruits (Fig. 3A), due to the strong reduction in the carotenoid content of the fruit pericarp (exocarp and mesocarp) (Fig. 3B). The T1 *pro35S::Cas9-Psy1* homozygous mutant #1 displays an insertion of one base in tomato fruit tissues and a bi-allelic mutation in leaves (both deletion and insertion of one base), resulting in a premature stop codon and a truncated protein (Fig. 3C). As expected with the 35 S promoter, no WT alleles were detected in both leaf and fruit tissues of *pro35S::Cas9-Psy1* (Fig. 3D).

We performed both phenotypic and genotypic screening of independent transgenic mutants *pronsLTP::Cas9-Psy1*. We excluded from our analysis the *pronsLTP::Cas9-Psy1 #2* mutant carrying the single +1 N mutation in all tissues analysed (Fig. S2). In the *pronsLTP::Cas9-Psy1* #4 and #5 mutants studied, ripe fruits displayed a reduced red pigmentation compared to the WT. The difference in the color of the fruit is even more obvious when the skin is peeled, revealing a clear orange-colored exocarp (Fig. 3A). These phenotypic results were confirmed by the significant reduction of carotenoid accumulation in ripe fruit pericarp of *pronsLTP::Cas9-Psy1* #4 and #5 (Fig. 3B). The proportion of KO mutations in the fruit exocarp of *pronsLTP::Cas9-Psy1* T1 mutants reached a high level of 71% to 82% (Fig. 3D), with a remarkable biological reproducibility between the three independent fruits of each plant (Fig. S3). Two mutations predominated, a base deletion and a codon deletion (respectively ~ 64% and ~ 21% of the total number of mutations, i.e. ~ 85%) leading respectively to a stop codon and a frameshift (Fig. 3C and Fig. S3). These results showed that the *nsLTP2* promoter can induce modifications in tomato fruit epidermis. *PronsLTP::Cas9-Psy1* #4 and #5 mutants showed 83% and 26% mutations in the fruit mesocarp respectively (Fig. 3C and D). The activity of the *nsLTP2* promoter in leaves, considering the entire leaf blade, including trichomes were examined and showed a rate of 5% (Fig. 3D).

### Overexpression of *SlMYB75* under the control of *pronsLTP* induces a specific coloration of the fruit epidermis

To provide a visual proof-of-concept that *pronsLTP* efficiently express genes in the epidermis at early stages of tomato fruit development, we choose to overexpress *SlMYB75* gene. This transcription factor promotes anthocyanin accumulation that led to a purple coloration of plant tissues [30]. It also regulates the phenylpropanoid pathway, whose derivatives such as p-coumaric acid are important compounds in the cuticle [11].

**Fig. 4:**
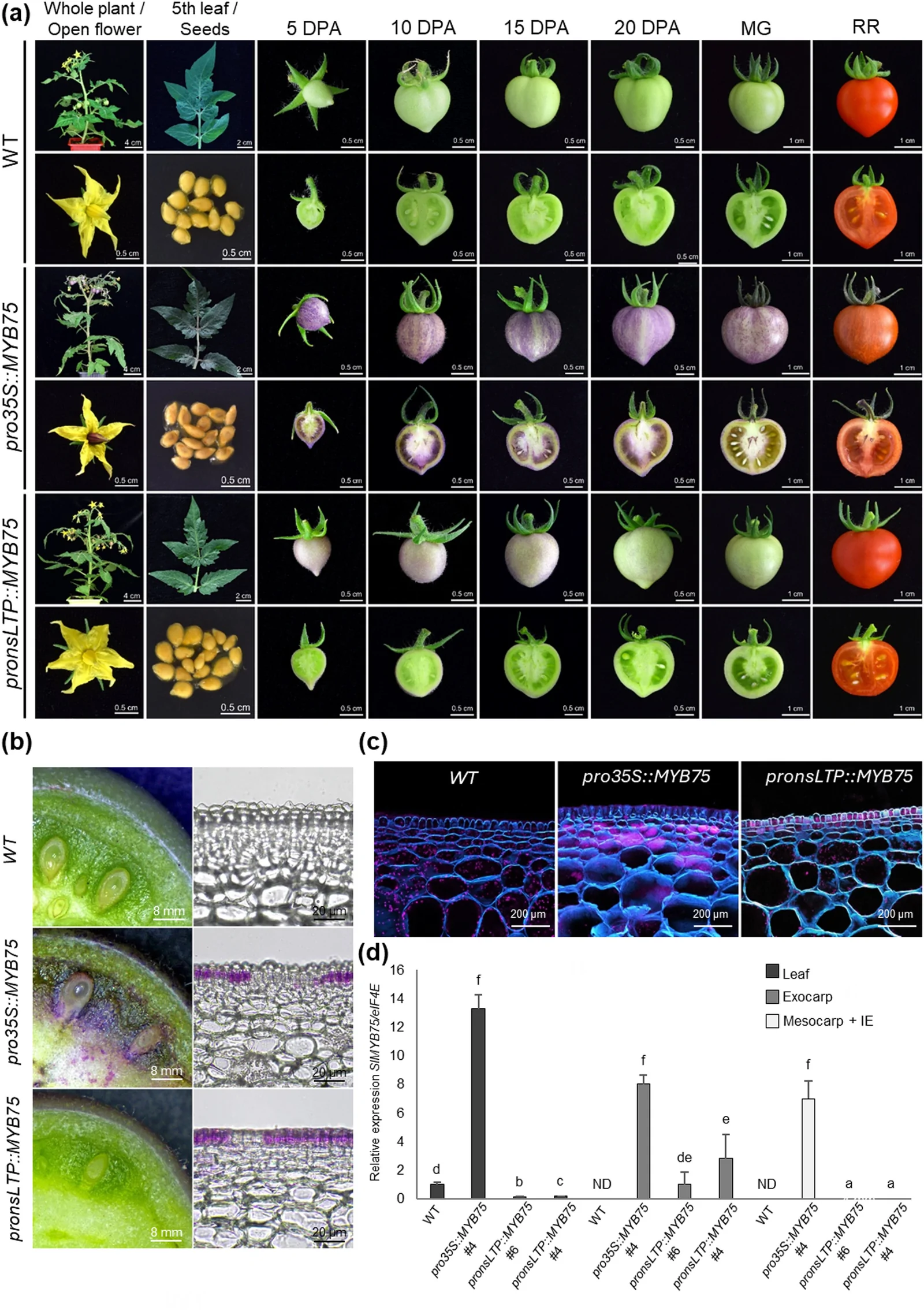
*pronsLTP*-driven expression of *SlMYB75* induces targeted accumulation of anthocyanins in fruit exocarp. **a** Anthocyanin accumulation in WT, *pronsLTP::MYB75* and *pronsLTP::MYB75* transgenic lines. Whole plant, leaf, flower, seeds and developing fruit from 5 DPA to RR stage are shown. DPA, Days Post Anthesis; MG, Mature Green; RR, Red Ripe. **b** Distribution of anthocyanin accumulation in 10 DPA fruit of WT, *pro35S::MYB75* and *pronsLTP::MYB75* transgenic lines. Left: cross-section of the whole fruit; right: vibratome cross-section of the pericarp under the optical microscope. **c** Confocal microscopy detection of anthocyanins (purple) after calcofluor white staining (blue) of vibratome cross-section of 10 DPA fruit pericarp of WT, *prons35S::MYB75* and *pronsLTP::MYB75*. Red dots in the cells are due to chloroplast autofluorescence. **d** RT-qPCR analysis of *SlMYB75* transcript abundance in leaf, fruit exocarp, and fruit mesocarp (including inner epidermis) of WT, *pro35S::MYB75* #4 and *pronsLTP::MYB75* #6 and #4. Values are mean ± SD (*n* = 3), with transcripts normalized to *eIF4E*. Lower-case letters indicate significant differences (*P* < 0.05; Kruskal-Wallis test with Tukey post-hoc test). ND, not detected

The *SlMYB75*-induced purple color pattern of *pronsLTP::MYB75* and *pro35S::MYB75* transformed plants was then scrutinized in the whole plant, leaf and open flower and throughout fruit development at 5 DPA, 10 DPA, 15 DPA, 20 DPA, mature green (MG; transition to ripening) and red ripe (RR; ripe fruit) stages (Fig. 4A). As expected, a purple coloration was observed in all *pro35S::MYB75* plant organs, while no color was detected in WT controls (Fig. 4A). No purple coloration was observed in the vegetative tissues, seeds and flower of *pronsLTP::MYB75* lines. In addition, a specific accumulation of pigments was observed in the early stages of fruit development from 5 DPA to 20 DPA, with a maximum from 5 DPA to 15 DPA (Fig. 4A). In the internal part of the *pronsLTP::MYB75* fruit, only the outer part of the pericarp displayed a purple color while all tissues, including pericarp, columella, placenta and locular tissue of *pro35S::MYB75* fruit were purple colored. (Fig. 4A). Light microscopy examination of the distribution of purple color in cross-sections of 20 DPA fruits indicated that in *pronsLTP::MYB75* fruits, the coloration was specifically observed in the outer epidermis, unlike in *pro35S::MYB75* fruits (Fig. 4B). Outer epidermis of both *pronsLTP::MYB75* and *pro35S::MYB75* fruit displayed a heterogeneous distribution of purple color (Fig. 4B), consistent with the variegated color of the fruit surface (Fig. 4A).

Confocal microscopy was used to analyze cross-sections of 10 DPA fruit, revealing purple pigmentation accumulation in the epidermal cell layers. The *prons35S::MYB75* plants showed a large accumulation of anthocyanins in various fruit tissues, including E2 and E3 sub-epidermal cell layers, but not E1 cell layer (Fig. 4C), in line with light microscopy observations (Fig. 4B). In contrast, in *pronsLTP::MYB75* fruit, the anthocyanin accumulation was specific to the epidermis (E1) and E2 sub-epidermal cell layers.

To validate the specific activity of *pronsLTP* in fruit tissues, we quantified the abundance of the *SlMYB75* transcript in leaves and two distinct parts of the fruit: the exocarp (composed of the outer epidermis and a few underlying layers of collenchyma) and the mesocarp (composed of collenchyma, parenchyma, vascular tissue and the inner epidermis) (Fig. 4D). In WT, *SlMYB75* expression was only detected in leaves. In the *pro35S::MYB75*, *SlMYB75* was found to be highly expressed in all the tissues while the *pronsLTP::MYB75* #6 and #4 lines showed a significant increase in *SlMYB75* transcripts in fruit exocarp specifically (Fig. 4D).

Taken together, these results show that *pronsLTP* is a promoter of choice for targeted modification in the tomato fruit epidermis during the early stages of development.

### Overexpression of *SlMYB75* under the control of *pronsLTP* modifies the lipid and phenolics composition in the tomato fruit cuticle

**Fig. 5:**
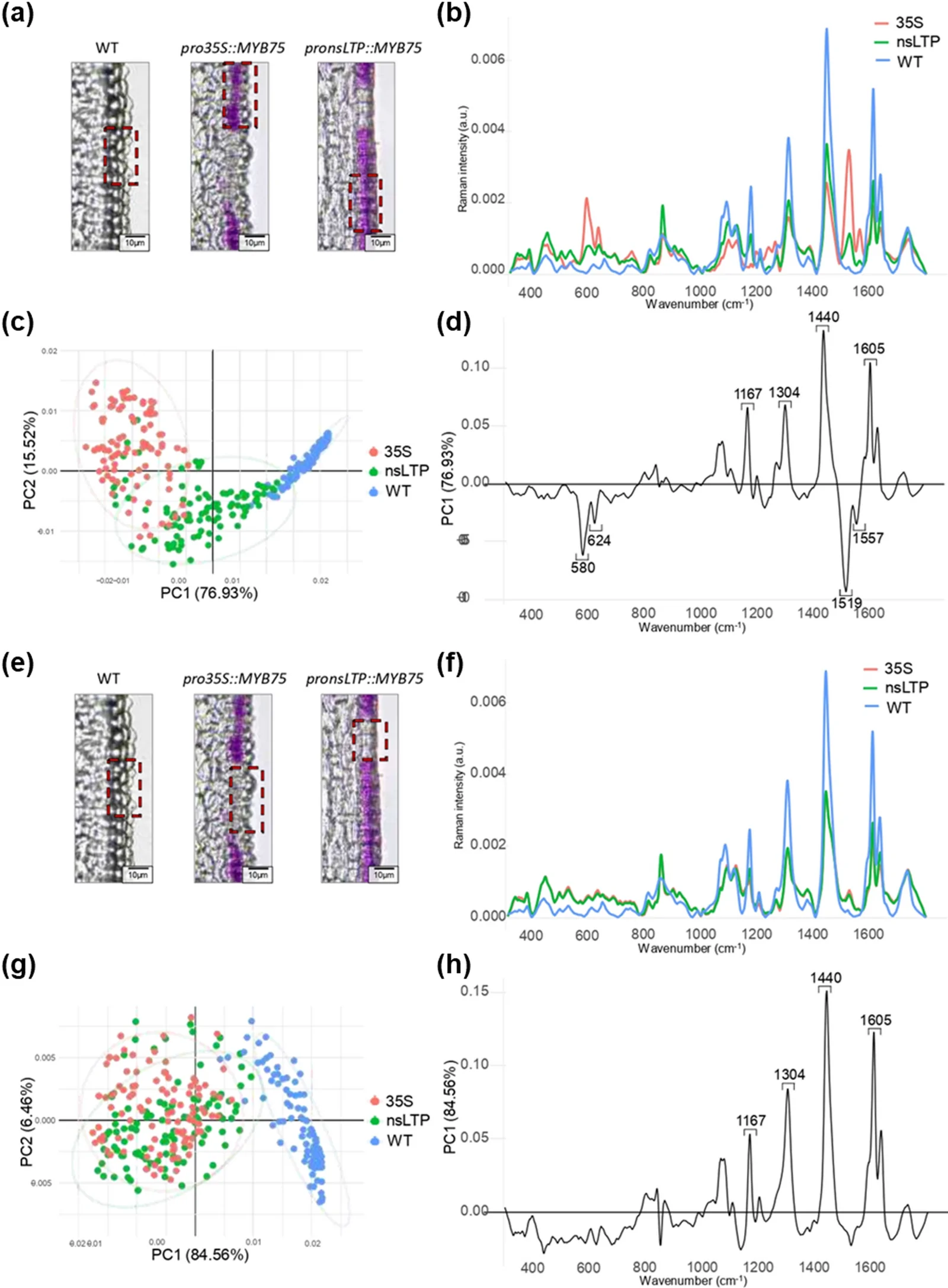
Raman spectroscopy analysis of the fruit cuticle composition of *SlMYB75* overexpressing fruits. **a** Areas of WT and purple epidermis analyzed by Raman spectroscopy in *SlMYB75* overexpressing lines. Photographs shown are from Fig. 3b. **b** Average spectra acquired on WT and purple areas of *pro35S::MYB75 (line #4)* and *pronsLTP::MYB75 (line #6).* Spectra were always taken between two adjacent epidermal cells. **C** Scatter plot of PCA analysis performed on spectra acquired from WT and purple areas of *pro35S::MYB75 #4* and *pronsLTP::MYB75 #6.* **d** PC1 loading of the PCA analysis shown in (**c**) discriminating the WT from the purple epidermis of *SlMYB75* overexpressing lines. **e** Areas of white epidermis analyzed by Raman spectroscopy in WT and *SlMYB75* overexpressing lines. Photographs shown are from Fig. 3B. **f** Average spectra acquired on WT and white epidermis areas of *pro35S::MYB75 #4* and *pronsLTP::MYB75 #6*. Spectra were always taken between two adjacent epidermal cells. **g** Scatter plot of PCA analysis performed on spectra acquired from WT and white epidermis areas of *pro35S::MYB75 #4* and *pronsLTP::MYB75 #6*. **h** PC1 loading of the PCA analysis shown in (**g**) discriminating the WT from the white epidermis of *SlMYB75* overexpressing lines

We further investigated the fruit cuticle of these *pro35S::MYB75* and *pronsLTP::MYB75* anthocyanin-accumulating lines. Raman spectroscopy is well adapted to assessing the chemical signature of tomato fruit cuticular layer [22, 23]. Anthocyanins, water-soluble compounds sequestered in the cell vacuole [31], were detected in the outer wall of epidermal cells. According to the high spatial resolution of RAMAN spectroscopy, we could address the differences in fruit surface color observed in *pronsLTP::MYB75* and *pro35S::MYB75* fruits (Fig. 4B). Consequently, we targeted both purple (Fig. 5A) and white areas (Fig. 5F) in MG fruit, i.e. before the accumulation of cuticular flavonoids associated with ripening, which could interfere with the spectral analysis.

Principal component analysis (PCA) from Raman spectral analyses shows the distribution of WT, *pronsLTP::MYB75* and *pro35S::MYB75* fruit cuticles samples in purple-colored areas (Fig. 5A and C). The Raman spectra acquired from purple areas of the cuticle of *pronsLTP::MYB75* and *pro35S::MYB75* fruits (Fig. 5A) highlighted specific bands at 580 cm^− 1^; 624 cm^− 1^ and 1557 cm^− 1^ assigned to flavonoids, while the 1519 cm^− 1^ band assigned to the ν(C-C) band of anthocyanins with or without glucoside derivation (Fig. 5B–D). This result confirms that the purple coloration observed in the fruit epidermis when *SlMYB75* is overexpressed is due to the accumulation of flavonoids, including anthocyanins. Interestingly, *pronsLTP::MYB75* and *pro35S::MYB75* fruit cuticles contain less lipids according to the specific bands of CH_2_ (τCH_2_ band at 1304 cm^− 1^ and δ(CH_2_) band at 1440 cm^− 1^) and less p-coumaric acid (ν(C = C) at 1605 cm^− 1^), compared to the WT fruit cuticle (Fig. 5D).

To refine this hypothesis, we analysed the cuticle of the white-colored areas. To this end, the cuticles were enzymatically isolated to remove any non-cutinized material. PCA clearly differentiated white-colored areas samples of WT from *pronsLTP::MYB75* and *pro35S::MYB75* fruits, with no difference between the lines overexpressing *SlMYB75* (Fig. 5G and S4). Raman spectra obtained from the white-colored areas of the fruits also demonstrated differences between the *SlMYB75* overexpressing lines and the WT (Fig. 5E–H). Notably, the spectra of *pronsLTP::MYB75* and *pro35S::MYB75* fruits indicate the same lower proportion of both lipids (1304 cm^− 1^, 1440 cm^− 1^) and p-coumaric acid (1167 cm^− 1^, 1605 cm^− 1^), compared to the WT. These results were confirmed by the HPLC analysis of p-coumaric acid released from enzymatically-isolated cuticles (Fig. S4). Altogether, these analyses of the fruit cuticle showed that, in addition to flavonoid accumulation in the vacuoles in the epidermal cells (purple area), *pronsLTP::SlMYB75* overexpression induced a reduction in the cutin polyester and the cutin-associated phenolic acid, i.e. p-coumaric acid, in the fruit cuticle.

### Targeted overexpression of *SlMYB75* in the epidermis modifies the properties of tomato fruit cuticle

**Fig. 6:**
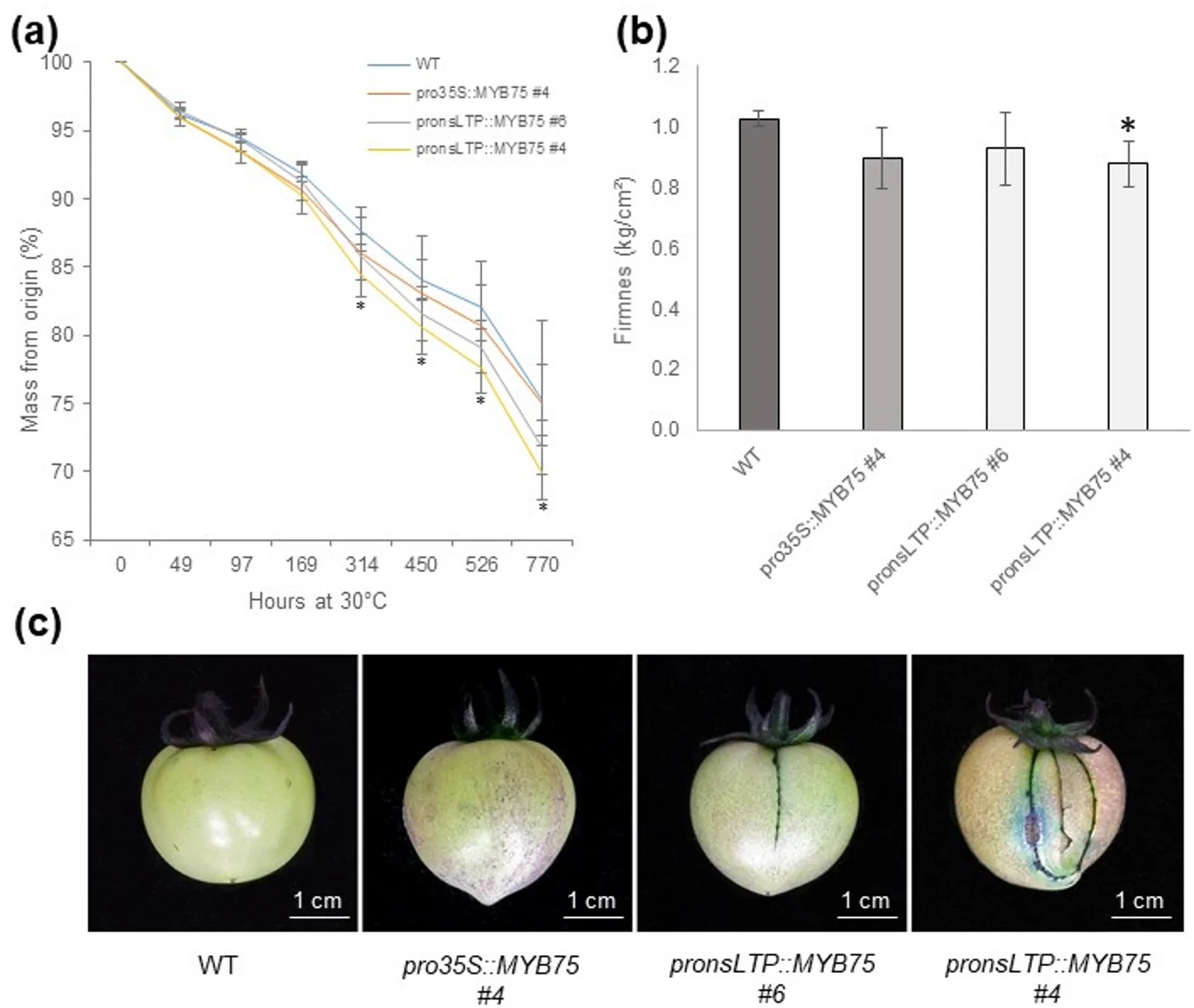
Cuticle properties of *SlMYB75* overexpressing fruits. **a** Water-loss in Red Ripe (RR) fruit of WT, *pro35S::MYB75 #4*, *pronsLTP::MYB75 #6* and *#4* during storage at 30 °C. Masses were recorded at time zero (T0) and after 49, 97, 169, 314, 450, 526 et 770 h. *, *P* < 0.05. **b** Penetrometry measurements on RR fruit. Values expressed in kg/cm² are mean ± SD (*n* = 5). *, *P* < 0.05; **, *P* < 0.01 (Student’s t test). **c** Permeability to toluidine blue of Mature Green fruits incubated with the dye during 16 h. Photographs of representative fruits are shown

We investigated whether overexpressing SlMYB75 modifies the properties of the cuticle. Water loss and firmness of RR fruit and permeability to toluidine blue (TB) of MG fruit (Fig. 6) were measured, which are all associated with cuticle integrity [32]. After 770 h of storage at 30 °C, a ~ 5.3 to 6.8% reduction in fruit fresh weight was detected in *pronsLTP::MYB75* fruits, with a significant reduction in *pronsLTP::MYB75#6.4* fruits (Fig. 6A). Conversely, *pro35S::MYB75* fruits exhibit no significant differences in water loss compared to WT (Fig. 6A). We also observed a significant reduction in fruit firmness in *pronsLTP::MYB75#6.4* fruits, while no change was observed for *pro35S::MYB75* (Fig. 6B). The most remarkable visual alterations of cuticle properties were observed after fruit immersion in TB, which revealed large cracks in up to 50% of *pronsLTP::MYB75* fruits (Fig. 6C) and was consistent with the alterations in water loss and firmness (Fig. 6A and B). Overall, these results demonstrate that the use of the *pronsLTP* for gene overexpression could induced a targeted changes in composition, leading to an alteration in the properties of the fruit’s epidermis.

## Discussion

### Identification of the *nsLTP2* promoter as a candidate for targeted cuticle modification

This study aimed to identify a promoter that could modify the composition of the tomato fruit cuticle at an early stage of development. This would provide an effective tool for deciphering the relationship between cuticle composition and properties. Among the promoters related to the cuticle described in the literature, none exhibited expression profiles with the spatial and/or temporal specificity expected to target the fruit and, even more so, the fruit epidermis at the early stage of development of the tomato fruit (i.e. high expression of cuticle-related genes).

Indeed, the *CRABS CLAW (CRC)*, *TOMATO PROLINE-RICH PROTEIN (TPRP)* and *POLYGALACTURONASE (PG)* promoters, although fruit-specific, failed to restrict gene expression to the epidermal layer in the Micro-Tom cultivar background [33]. We also excluded the widely used *PHOSPHOENOLPYRUVATE CARBOXYLASE* (*PPC2*) promoter targeting early stages of fruit development because it drives a strong expression in mesocarp cells and is not active in epidermal cells [33–35]. Similarly, the E8 promoter, widely used for ripening-specific expression, lacks activity during the early fruit development stages critical for cuticle formation [36]. Other promoters, such as those from *ECERIFERUM 6* (*SlCER6)*, gene encoding a VLCFA elongase β-ketoacyl‐CoA synthase involved in wax biosynthesis, and *CHALCONE SYNTHASE 1* (*SlCHS1*), gene encoding a chalcone synthase enzyme involved in flavonoid biosynthesis, have been reported to drive gene expression in fruit exocarp [3]. However, the *SlCER6* gene is expressed in both leaves and fruit, and its mutation has considerable effects on leaf wax load and composition [37]. The *SlCHS1* gene is also strongly expressed in flower buds according to the Tomato eFP browser Rose Lab Atlas (http://bar.utoronto.ca). Recently, alterations in fruit cuticle properties have also been demonstrated in fruit expressing the wax-regulating *MYB31* transcription factor (TF) under the control of the exocarp-preferential *PATHOGENESIS RELATED PROTEIN 10* (*SlPR10*) promoter [38]. However, the *SlPR10* promoter is also active in other plant organs, such as the leaf and stem. This was demonstrated by analysing transgenic plants that overexpress the *SlANT1* MYB transcription factor (TF), which promotes anthocyanin accumulation [38].

According to an in-depth study of gene expression in tomato, we selected the promoter of the *non-specific Lipid Transfer Protein 2* (*pronsLTP*) gene that (i) shows strong preferential expression in the outer epidermis compared to other fruit tissues and (ii) is active in the early stages of development, when most of the cuticle genes are expressed [3, 25]. Green Fluorescence assay confirmed the spatial expression pattern of the *nsLTP2* promoter, showing no detectable signal in root, leaf and flower. Although the various in silico tools for spatial expression analysis had not predicted *nsLTP2* expression elsewhere than in the fruit epidermis, our study showed that the *nsLTP2* promoter is also active in specialized cells, the leaf trichomes. Several studies indicated a link between nsLTP-type lipid transporters and trichomes. For example, the nsLTP McLTPII.9 protein from *Mentha canadensis* is involved in glandular trichome density and the metabolism of volatile compounds [39]. In tobacco, it has been shown that a *nsLTP* appears to be required for lipid secretion from glandular trichomes, indicating that *nsLTPs* may play a role in essential oil secretion or defense mechanisms [40]. However, trichomes are highly specialized cells that turn out to be non-essential for plant growth and development [41], and mutation of key genes involved in trichome biosynthesis have no consequences for other vegetative and reproductive tissues, as well as plant physiology [42]. For these reasons, the expression of the *nsLTP* promoter in trichomes was finally not an obstacle in the choice of *nsLTP* to target expression in the fruit epidermis. It is even conceivable that *pronsLTP* could be used for metabolite production in tomato trichomes [43, 44].

### The *nsLTP* promoter as a tool for tissue-specific knockout for functional studies of tomato fruit cuticle

According to our present data, the *nsLTP2* promoter efficiently drives knockout mutations in the tomato fruit using the CRISPR-TSKO approach. The pattern and level of expression of *pronsLTP* in tomato fruit epidermis at the earliest stages of development are key assets for ensuring early and sufficient expression of the CRISPR/Cas9 machinery to induce a high mutation rate (up to 82%). We designed a single specific guide and observed five types of mutations - two of which represented 85% of all the total mutations. A limitation of tissue-specific gene edition is the generation of independent *de novo* mutations, producing chimeric tissues [45]. Remarkably, our study found that the type and frequency of these mutations were highly consistent both within and between the two independent lines of the same mutant fruit. These results highlight the importance not only of the promoter but also of the choice of sgRNA used to optimise results. In fact, several studies have shown that the guide sequence influences the type of mutations, promoting specific types of mutations [46, 47]. A screening method and an efficiency test for sgRNAs in protoplasts would make it possible to select the most interesting guides, as shown in *Arabidopsis thaliana* and Sorghum studies [45, 48]. As demonstrated by Bollier et al. [49], multiplex CRISPR approaches for gene editing in specific plant tissues can be used to simultaneously inactivate multiple genes and target redundant gene families. Furthermore, the detection of a heterozygous mutant during the screening stage highlights the need to optimise the transformation and/or regeneration process to improve efficiency and limit the production of stable transformants.

CRISPR-TSKO is a powerful tool with numerous limitations [45, 50] and one of the main restrictions is the specificity of the promoter. For this reason, we have chosen to precisely study expression in different tissues, both vegetative by sequencing leaves and in fruit, with two distinct tissues, the exocarp and mesocarp. Whereas exocarp cells are highly mutated, we found variable percentages of mutations in the mesocarp of the fruit. The mutations in the mesocarp can be explained by the fact that during early fruit development, the mesocarp cell layers originate from the exocarp. Indeed, the E2 and E3 layers of subepidermal cells of the exocarp undergo periclinal divisions that generate the cells of the mesocarp [4]. Low *pronsLTP* expression in these cells may be sufficient to induce mutations in mesocarp cells. Nevertheless, we have shown a low proportion of mutations in leaf tissue, likely due to the *pronsLTP* limited expression in trichomes. These results confirm that the activity of the promoter is strongly limited to fruit, which represents a significant advance in limiting the pleiotropic effects generally associated with the usual constitutive promoters such as the 35 S promoter.

This strategy is particularly relevant in light of recent developments in CRISPR-edited tomatoes, such as those enriched in γ-aminobutyric acid (GABA) [51]. This study has indeed indicated that high levels of GABA in vegetative tissues, particularly leaves, can reduce plant size and yield. This led the scientists to select a fruit-specific gene to mitigate the pleiotropic agronomic effects. In this context, the *nsLTP2* promoter is a useful tool for driving efficient, early modification in tomato fruit without affecting vegetative growth.

### Overexpression under the control of *pronsLTP*, targeting epidermis, is a molecular tool to explore the link between cuticle composition and properties

Using anthocyanins as a case study, we demonstrated that overexpression of the *SlMYB75* gene with the *nsLTP2* promoter resulted in a modification of cuticle composition and properties. In the fruit cuticle, Raman spectroscopy showed a reduction in cutin and the cutin-associated phenolic acid, p-coumaric acid. As p-coumaric acid is a precursor of flavonoids, including anthocyanins, this result likely indicates that the phenypropanoid flux is partly boosted towards the formation of anthocyanins, at the expense of the p-coumaric acid accumulation in the fruit cuticle. These cuticle chemical modifications probably impacted the cuticle structure that most likely account for the alteration in water loss and the firmness of the *pronsLTP::MYB75* fruits, according to the previous results in [1].

The use of the *nsLTP2* promoter would be a significant asset to pinpoint the role of the different components of the cuticle. This molecular tool could be used to decipher, in particular, the role of polysaccharides and their impact on properties that remain unclear. In tomato, recent studies have shown that the spatial distribution and evolution of esterified pectin, crystalline cellulose and acetylated xyloglucan during fruit development affect the local mechanical properties of the cuticle [23]. However, it remains difficult to determine the individual contribution of each component to these properties, as these polymers are essential and ubiquitous in the plant, making their systemic modification potentially lethal. In this regard, targeted modification of these components associated with the cuticle is therefore an effective approach to overcoming these limitations. Among the genes of interest are those involved in the biosynthesis or assembly of cuticle-associated polysaccharides, such as *xyloglucan endotransglycosylase/hydrolase* (*XTH*) genes which are associated with xyloglucan remodeling [52], and *pectin methyl esterase* (*PME*) genes, which are involved in pectin modification [53].

Targeted overexpression of these enzymes would induce localised changes in the composition of the cuticle structure. A cross talk between cutin and cutin-embedded polysaccharide deposition has been highlighted [15] in tomato fruit cuticule. Accordingly, a modification of one of the cuticle components (e.g. polysaccharide) could lead to a change in the spatial distribution of all cuticle compounds. These approaches are now more accessible thanks to the high spatial resolution of chemical mapping analyses achieved by microspectroscopy [54]. The changes in composition are likely to impact the properties of the cuticle, such as its mechanical behaviour, permeability to water and chemicals, and susceptibility to enzymatic hydrolysis by pathogens. Finally, beyond its agronomic interest, the cuticle of the tomato fruit is a remarkable source of inspiration for the design of composites, combining lipids, phenolics and polysaccharides in a thin, multifunctional barrier.

## Conclusions

This study successfully validated the utilization of the *nsLTP2* promoter for the scientific community as a promoter suitable for fruit cuticle engineering in tomato. This promoter enables targeted overexpression of genes of interest in the epidermis of tomato fruit to decipher how these compounds modulate cuticle architecture and properties. The CRISPR system can also be used in association with the nsLTP2 promoter to target genes of interest in tomato fruit. This work also raises apparent discrepancies between the chemical composition of the cuticle and its properties, providing a framework for exploring these relationships. To this end, in-depth investigations will be carried out in the near-future using up-to-date technologies developed for correlative multimodal imaging approach and the plant material generated here. In addition to fundamental research, the *nsLTP2* promoter could also serve as a valuable tool for crop improvement strategies aimed at enhancing fruit surface properties such as shelf life, resistance to cracking, pathogen tolerance and postharvest quality, whilst minimising pleiotropic effects on vegetative development.

## Methods

### Plant material and culture conditions

Tomato (*Solanum lycopersicum* cv. Micro-Tom) plants were grown in a greenhouse as described in [7]. Flowers were regularly vibrated to ensure even pollination and tagged at anthesis.

### Gene expression analysis

Gene expression data (Reads Per Million mapped reads; RPM) for the different tomato fruit tissues were extracted from [24]. We selected the 500 most variable genes across five fruit tissues: outer epidermis, collenchyma, parenchyma, vascular tissue, and inner epidermis, at 10 days post anthesis (10 DPA). Genes must be expressed at least in the outer epidermis, our tissue of interest. Hierarchical clustering was performed and heatmap were obtained from z-scores (centred-reduced data) calculated from RPM values using Rstudio software and the ‘pheatmap (v1.0.12)’ package. Dot plot was generated using the ‘ggplot2’ (v3.5.1) package, based on log-transformed expression values (log_10_(RPM + 1)). Gene expression data (normalized values) for leaf, root and flower organs were extracted from TomExpress database [27]. I*n-silico* analysis of the *nsLTP2* promoter sequence was performed using the PlantCARE database [55].

### Overexpression of *NLS-GFP* and *SlMYB75* and plant transformation

Overexpression vectors were constructed by GoldenGate^®^ cloning of amplified coding sequence of *SlMYB75* (*Solyc10g080610*) and Green Fluorescent Protein in translational fusion to a nuclear localization signal (*NLS-GFP* from the pXK7S*NF2 vector [56]), (see Table S4 for gene specific primers). Open reading frames of *Sl*MYB75 or NLS-GFP were placed under the control of either the *CAMV 35 S* promoter (2 × 35 S duplicated promoter) or a 2498 bp DNA fragment located directly upstream of the ATG codon of the *nsLTP2* gene (*pronsLTP*) and followed by OCS terminator and HSP18.2 respectively. A *BpiI* enzymatic restriction site was removed from the *pronsLTP* sequence by 1-bp substitution (C to G) to facilitate cloning. To generate transgenic plants, constructs were transformed into *Agrobacterium tumefaciens* (strain C58C1), and then into tomato (Micro-Tom cv) as previously described [33] plantlets were further checked for ploidy level by flow-cytometry analysis and polyploid plants were discarded. Between 12 and 15 independently *pronsLTP::MYB75* and *pro35S::MYB75* transformed plants were generated for each *pro::MYB75* transgene construct. Two T2 representative lines were selected for each construct.

### CRISPR/Cas9-engineered *psy1*

CRISPR/Cas9 mutagenesis of *SlPSY1* (*Solyc03g031860*) was done as previously described [57] using a single sgRNA guide to induce point mutations in the *PSY1* sequence designed with CRISPOR (http://crispor.gi.ucsc.edu/crispor.py) (Table S3). Resulting mutations were detected in leaves and fruit tissues (exocarp or mesocarp plus inner epidermis) from T1 lines by PCR and Sanger sequencing. Mutations frequency was analyzed with ICE Analysis, 2019, v3.0, from Synthego.

### Measurement of fruit skin properties

Fruit skin properties were measured as described in [58]. For measurements of cuticle permeability to stain, MG fruits collected from WT, *pro35S::MYB75* and *pronsLTP::MYB75* T2 plants (3 fruits per plant) were placed in 0.1% toluidine blue solution for 16 h. After water rinsing, fruit surface staining was visually assessed, and photographs were taken under standardized conditions as done in [25]. For water loss measurements, red ripe (RR) fruits harvested from WT, *pro35S::MYB75* and *pronsLTP::MYB75* T2 plants (3 fruits per plant) were stored at 30 °C in a dry oven for 770 h. Water loss was calculated as a percentage of weight loss. Fruit firmness was assessed by penetrometry using the Fruit Texture Analyser (GüSS, South Africa) equipped with an 8-mm diameter probe, as previously described [57].

### Measurement of carotenoid and p-coumaric content

Carotenoid concentration was measured from 400 mg of frozen fruit pericarp tissue from T1 plants, ground for 30 s using a Dangoumo milling system (Prolabo) at nominal frequency. After acetone extraction [58], the absorbance of a 50 µL test sample was measured at 474 nm against an acetone blank with an Amersham Ultrospec 3100 Pro (Amersham Bioscience) using a quartz cuvette. The carotenoid concentration, expressed in µg/mg Fresh Weight (FW), was calculated as described in [59].

In addition, the amount of p-coumaric acid was measured after alkaline hydrolysis of cutin. Briefly, 5 mg of isolated cuticle was treated overnight in KOH/methanol (4% w: w). The supernatant was analysed by high-performance liquid chromatography (HPLC)- with UV detection at 310 nm on an Acquity ARC system (Waters, USA) equipped with a C18 column (250 × 4.6 mm, 5 μm) as previously describe [60]. Quantification of released p-coumaric was conducted on at least 5 replicates and according an external calibration with a p-coumaric standard.

### Macro and microscopic analysis

Macroscopic observations of tissues from WT and *pronsLTP::NLS-GFP* plants were carried out on an Axiozoom V16 (Zeiss) using the GFP fluorescence acquisition mode at 496 nm. GFP and white light pictures (in grey level mode) were overlaid to have both signals. For confocal microscopy observations, 3 independent 5 DPA fruits were used for each genotype. Fine Sect. (120 μm) were obtained using a vibrating blade microtome (Microm HM 650 V, Thermo Scientific), stained 30 s in 0.05% calcofluor white (Sigma-Aldrich), washed twice for 5 min with PBS 1X, mounted on a glass slide in PBS 1X and imaged using a laser scanning microscope in confocal mode (Zeiss LSM 880 AIRYSCAN). Photographs of anthocyanin accumulation in plant organs and during fruit development of WT, *pronsLTP::MYB75* and *pro35S::MYB75* T2 lines were taken under standardized conditions. Macroscopic observations in white light were performed with an Axiozoom V16 (Zeiss) on longitudinal sections of 10 DPA fruit. Thick Sect. (120 μm) of fresh fruit were obtained with a Microm HM 650 V vibratome (Thermo Scientific) and imaged with an AxioImager microscope (Zeiss). For confocal acquisition, 150 μm thick sections of 5 DPA fruit pericarp were stained with calcofluor white and observed at 405 nm and 594 nm for anthocyanin autofluorescence with a Zeiss LSM 880 AIRYSCAN. All these experiments were realized at the Bordeaux Imaging Center (http://www.bic.u-bordeaux.fr/).

### RT-qPCR gene expression analysis

Twelve 1 cm diameter discs of epidermal peel were isolated from six 20 DPA fruits collected from three independent WT, *pronsLTP::MYB75* and *pro35S::MYB75* T2 plants and carefully scratched with a scalpel blade (exocarp samples) as previously described [28]. Two disks per fruit were collected in distinct pools and three pools were made in order to obtain three biological replicates. Pericarp samples, consisting of the whole pericarp minus the peel, were prepared in the same way. Samples were ground in liquid nitrogen and stored at −80 °C until RNA extraction. RNA was extracted using the RNA purification from Plant and Fungi Nucleospin kit (Macherey-Nagel). Total RNA integrity and concentration were assessed using an Agilent 2100 Bioanalyzer with RNA Nano Chip (Agilent Technologies). Reverse transcription was carried out using 500 ng of total RNA and Invitrogen SuperScript IV reverse transcriptase following the manufacturer instructions. qRT-PCR was performed with *SlMYB75* specific primers (Table S4) and GoTaq qPCR Master Mix (Promega) using the CFX96 Touch Real-Time system (Bio-Rad). Three biological and three technical replicates were performed per point. *EiF4e* was used as reference gene to calculate the relative expression changes according to the ΔΔCT method.

### Raman spectroscopy

The cuticles of 3 MG fruits collected from WT, *pro35S::MYB75* and *pronsLTP::MYB75* plants were hand isolated with a spatula and dried before Raman analysis. When indicated, cuticles were also submitted to enzymatic purification in baths containing 0.5% cellulase (*Aspergillus sp*., EC 3.2.1.4, Sigma-Aldrich) and 0.5% pectinase (*Aspergillus aculateus*., EC 3.2.1.15, Sigma-Aldrich) during seven days and dried at room temperature. Both hand-isolated and digested cuticles were then used for Raman analysis using an inVia confocal Raman microspectrometer (Renishaw) with a 785 nm laser and a 1200 L/mm grating. Prior to use, the instrument was calibrated using a silicon reference. Spectra were acquired from two independent tomato fruits of each plant on area situated at the junction of three epidermal cells visible with the Raman optical on cuticles before and after enzymatic digestion. This area corresponds to the central furrow of the cuticle commonly observed in cuticle cross-section images.

Measurements were conducted within the range of 300 to 1800 cm^− 1^. The laser power was set at 100% and the sample was exposed for six seconds with two accumulations, using a 50× objective (Leica, NA = 0.75). Z-stack mode was employed to scan the cuticle central furrows in depth, resulting in five acquired spectra on the same location with a 5 μm step interval in the z-axis. This enabled the entire cuticle depth to be scanned. For each *pro35S::MYB75* and *pronsLTP::MYB75* fruits, 14 locations were targeted i.e. seven in white cuticle areas and seven in purple areas. Accordingly, seven different random locations were acquired on three pieces of cuticle from WT, *pro35S::MYB75* and *pronsLTP::MYB75* cuticles. The Raman spectra was pre-processed using the Spectragryph software (optical spectroscopy software, version 1.2.16.1, 2022) with the following steps: smoothing, baseline correction, and area normalization. The average spectra for each sample and acquisition area were plotted for peak attribution. The individual spectra were used for band integration and statistical analysis, with the ANOVA test performed in the Rstudio software.

### Statistical analysis

Statistical analyses were performed using RStudio software (www.r-studio.com). For pairwise comparisons of *nsLTP2* gene expression in mutants and for cuticle properties of SlMYB75 overexpressing fruits (water loss and penetrometry measurements), Student’s t-tests were applied. For multi-group comparisons, Kruskal–Wallis tests were used, followed by a Tukey’s post hoc test. This approach was applied to carotenoid content measurements SlPSY1 mutants and RT-qPCR analyses of SlMYB75 transcript abundance in SlMYB75 transformants tissues. All statistical analyses were performed using a significance threshold of *p* < 0.05 or *p* < 0.01.

## Supplementary Information


Supplementary Material 1.



Supplementary Material 2.


## Data Availability

The expression data from laser-microdissected fruit tissues were extracted from Supplementary Data 3 of [24]. RNA sequencing data were extracted from the tomato RNA-seq web platform (TomExpress) [27]. All other data are are included in the manuscript and the supporting materials. The materials (e.g. vectors) that support the findings of this study are available on request from the corresponding author.

## References

[1] Petit J, Bres C, Mauxion JP, Bakan B, Rothan C. Breeding for cuticle-associated traits in crop species: traits, targets, and strategies. J Exp Bot 9 nov. 2017;68(19):5369–87. 10.1093/jxb/erx341. 29036305

[2] Lemaire-Chamley M, Petit J, Garcia V, Just D, Baldet P, Germain V, et al. Changes in transcriptional profiles are associated with early fruit tissue specialization in tomato. Plant Physiol oct. 2005;139(2):750–69. 10.1104/pp.105.063719. 16183847 PMC1255993

[3] Mintz-Oron S, Mandel T, Rogachev I, Feldberg L, Lotan O, Yativ M, et al. Gene Expression and Metabolism in Tomato Fruit Surface Tissues. Plant Physiol juin. 2008;147(2):823–51. 10.1104/pp.108.116004. 18441227 PMC2409049

[4] Renaudin JP, Deluche C, Cheniclet C, Chevalier C, Frangne N. Cell layer-specific patterns of cell division and cell expansion during fruit set and fruit growth in tomato pericarp. J Exp Bot 1 mars. 2017;68(7):1613–23. 10.1093/jxb/erx. 28369617 PMC5444452

[5] Shi JX, Adato A, Alkan N, He Y, Lashbrooke J, Matas AJ, et al. The tomato SlSHINE3 transcription factor regulates fruit cuticle formation and epidermal patterning. New Phytol janv. 2013;197(2):468–80. 10.1111/nph.12032. 23205954

[6] Matas AJ, Yeats TH, Buda GJ, Zheng Y, Chatterjee S, Tohge T, et al. Tissue- and cell-type specific transcriptome profiling of expanding tomato fruit provides insights into metabolic and regulatory specialization and cuticle formation. Plant Cell 1 nov. 2011;23(11):3893–910. 10.1105/tpc.111.091173. 22045915 PMC3246317

[7] Rothan C, Diouf I, Causse M. Trait discovery and editing in tomato. Plant J janv. 2019;97(1):73–90. 10.1111/tpj.14152 . PubMed PMID: 30417464. 30417464

[8] España L, Heredia-Guerrero JA, Segado P, Benítez JJ, Heredia A, Domínguez E. Biomechanical properties of the tomato (Solanum lycopersicum) fruit cuticle during development are modulated by changes in the relative amounts of its components. New Phytol mai. 2014;202(3):790–802. 10.1111/nph.12727 . PubMed PMID: 24571168. 24571168

[9] Girard AL, Mounet F, Lemaire-Chamley M, Gaillard C, Elmorjani K, Vivancos J, et al. Tomato GDSL1 is required for cutin deposition in the fruit cuticle. Plant Cell. 2012;24(7):3119–34. 10.1105/tpc.112.101055. 22805434 PMC3426136

[10] Moreira CJS, Bento A, Pais J, Petit J, Escórcio R, Correia VG, et al. An ionic liquid extraction that preserves the molecular structure of cutin shown by nuclear magnetic resonance. Plant Physiol. 2020;184(2):592–606. 10.1104/pp.20.01049. 32788301 PMC7536654

[11] Reynoud N, Petit J, Bres C, Lahaye M, Rothan C, Marion D, et al. The Complex Architecture of Plant Cuticles and Its Relation to Multiple Biological Functions. Front Plant Sci. 2021;12:782773. 10.3389/fpls.2021.782773 . PubMed PMID: 34956280; PubMed Central PMCID: PMC8702516. 34956280 PMC8702516

[12] Yeats TH, Howe KJ, Matas AJ, Buda GJ, Thannhauser TW, Rose JKC. Mining the surface proteome of tomato (*Solanum lycopersicum*) fruit for proteins associated with cuticle biogenesis. J Exp Bot. 2010;61(13):3759–71. 10.1093/jxb/erq194. 20571035 PMC2921210

[13] Petit J, Bres C, Reynoud N, Lahaye M, Marion D, Bakan B, et al. Unraveling Cuticle Formation, Structure, and Properties by Using Tomato Genetic Diversity. Front Plant Sci. 2021;12:778131. 10.3389/fpls.2021.778131 . PubMed PMID: 34912361; PubMed Central PMCID: PMC8667768. 34912361 PMC8667768

[14] Elejalde-Palmett C, Martinez San Segundo I, Garroum I, Charrier L, De Bellis D, Mucciolo A, et al. ABCG transporters export cutin precursors for the formation of the plant cuticle. Curr Biol. 2021;31(10):2111-2123.e9. 10.1016/j.cub.2021.02.056. 33756108

[15] Philippe G, Geneix N, Petit J, Guillon F, Sandt C, Rothan C, et al. Assembly of tomato fruit cuticles: a cross-talk between the cutin polyester and cell wall polysaccharides. New Phytol. 2020;226(3):809–22. 10.1111/nph.16402. 31883116

[16] Bernard A, Joubès J. *Arabidopsis* cuticular waxes: advances in synthesis, export and regulation. Prog Lipid Res. 2013;52(1):110–29. 10.1016/j.plipres.2012.10.002. 23103356

[17] Fich EA, Segerson NA, Rose JKC. The Plant Polyester Cutin: Biosynthesis, Structure, and Biological Roles. Annu Rev Plant Biol. 2016;67:207–33. 10.1146/annurev-arplant-043015-111929. 26865339

[18] Philippe G, Sørensen I, Jiao C, Sun X, Fei Z, Domozych DS, et al. Cutin and suberin: assembly and origins of specialized lipidic cell wall scaffolds. Curr Opin Plant Biol. 2020;55:11–20. 10.1016/j.pbi.2020.01.008. 32203682

[19] Philippe G, Gaillard C, Petit J, Geneix N, Dalgalarrondo M, Bres C, et al. Ester Cross-Link Profiling of the Cutin Polymer of Wild-Type and Cutin Synthase Tomato Mutants Highlights Different Mechanisms of Polymerization. Plant Physiol. 2016;170(2):807–20. 10.1104/pp.15.01620. 26676255 PMC4734573

[20] Edqvist J, Blomqvist K, Nieuwland J, Salminen TA. Plant lipid transfer proteins: are we finally closing in on the roles of these enigmatic proteins? J Lipid Res août. 2018;59(8):1374–82. 10.1194/jlr.R. 29555656 PMC6071764

[21] González Moreno A, Domínguez E, Mayer K, Xiao N, Bock P, Heredia A, et al. 3D (x-y-t) Raman imaging of tomato fruit cuticle: Microchemistry during development. Plant Physiol. 2023;191(1):219–32. 10.1093/plphys/kiac369. 35972400 PMC9806558

[22] Reynoud N, Geneix N, Petit J, D’Orlando A, Fanuel M, Marion D, et al. The cutin polymer matrix undergoes a fine architectural tuning from early tomato fruit development to ripening. Plant Physiol. 2022;190(3):1821–40. 10.1093/plphys/kiac392. 36018278 PMC9614491

[23] Reynoud N, Geneix N, D’Orlando A, Petit J, Mathurin J, Deniset-Besseau A, et al. Cuticle architecture and mechanical properties: a functional relationship delineated through correlated multimodal imaging. New Phytol. 2023;238(5):2033–46. 10.1111/nph.18862. 36869436

[24] Shinozaki Y, Nicolas P, Fernandez-Pozo N, Ma Q, Evanich DJ, Shi Y, et al. High-resolution spatiotemporal transcriptome mapping of tomato fruit development and ripening. Nat Commun. 2018;9(1):364. 10.1038/s41467-017-02782-9. 29371663 PMC5785480

[25] Petit J, Bres C, Just D, Garcia V, Mauxion JP, Marion D, et al. Analyses of tomato fruit brightness mutants uncover both cutin-deficient and cutin-abundant mutants and a new hypomorphic allele of GDSL lipase. Plant Physiol. 2014;164(2):888–906. 10.1104/pp.113.232645. 24357602 PMC3912114

[26] Isaacson T, Kosma DK, Matas AJ, Buda GJ, He Y, Yu B, et al. Cutin deficiency in the tomato fruit cuticle consistently affects resistance to microbial infection and biomechanical properties, but not transpirational water loss. Plant J oct. 2009;60(2):363–77. 10.1111/j.1365-313X. 19594708

[27] Zouine M, Maza E, Djari A, Lauvernier M, Frasse P, Smouni A, et al. TomExpress, a unified tomato RNA-Seq platform for visualization of expression data, clustering and correlation networks. Plant J. 2017;92(4):727–35. 10.1111/tpj.13711. 28873253

[28] Petit J, Bres C, Mauxion JP, Tai FWJ, Martin LBB, Fich EA, et al. The Glycerol-3-Phosphate Acyltransferase GPAT6 from Tomato Plays a Central Role in Fruit Cutin Biosynthesis. Plant Physiol juin. 2016;171(2):894–913. 10.1104/pp.16.00409. PMC4902622 27208295

[29] Cao X, Du R, Xu Y, Wu Y, Ye K, Ma J, et al. Phytoene synthases 1 modulates tomato fruit quality through influencing the metabolic flux between carotenoid and flavonoid pathways. Hortic Plant J. 2024;10(6):1383–97. 10.1016/j.hpj.2022.09.015.

[30] Jian W, Ou X, Sun L, Chen Y, Liu S, Lu W, Yang X, Zhao Z, Li Z. Characterization of anthocyanin accumulation, nutritional properties, and postharvest attributes of transgenic purple tomato. Food Chem. 2023;408: 135181. 10.1016/j.foodchem.2022.135181. 36525727

[31] Gould KS. Nature′s Swiss Army Knife: The Diverse Protective Roles of Anthocyanins in Leaves. Biomed Res Int. 2004;2004(5):415423. 10.1155/S1110724304406147. PMC1082902 15577195

[32] Tanaka T, Tanaka H, Machida C, Watanabe M, Machida Y. A new method for rapid visualization of defects in leaf cuticle reveals five intrinsic patterns of surface defects in Arabidopsis. Plant J janv. 2004;37(1):139–46. 10.1046/j.1365-313x. 14675439

[33] Fernandez AI, Viron N, Alhagdow M, Karimi M, Jones M, Amsellem Z, et al. Flexible tools for gene expression and silencing in tomato. Plant Physiol déc. 2009;151(4):1729–40. 10.1104/pp.109.147546. 19812183 PMC2785966

[34] Ernesto Bianchetti R, Silvestre Lira B, Santos Monteiro S, Demarco D, Purgatto E, Rothan C, et al. Fruit-localized phytochromes regulate plastid biogenesis, starch synthesis, and carotenoid metabolism in tomato. J Exp Bot. 2018;69(15):3573–86. 10.1093/jxb/ery145. 29912373 PMC6022544

[35] Guillet C, Aboul-Soud MAM, Le Menn A, Viron N, Pribat A, Germain V, et al. Regulation of the fruit-specific PEP carboxylase SlPPC2 promoter at early stages of tomato fruit development. PLoS ONE. 2012;7(5):e36795. 10.1371/journal.pone.0036795. 22615815 PMC3355170

[36] Nambeesan S, Datsenka T, Ferruzzi MG, Malladi A, Mattoo AK, Handa AK. Overexpression of yeast spermidine synthase impacts ripening, senescence and decay symptoms in tomato. Plant J. 2010;63(5):836–47. 10.1111/j.1365-313X. 20584149

[37] Vogg G, Fischer S, Leide J, Emmanuel E, Jetter R, Levy AA, et al. Tomato fruit cuticular waxes and their effects on transpiration barrier properties: functional characterization of a mutant deficient in a very-long-chain fatty acid beta-ketoacyl-CoA synthase. J Exp Bot. 2004;55(401):1401–10. 10.1093/jxb/erh149. 15133057

[38] Ruan XM, Xiong X, Li JF. Identification and application of an exocarp-preferential promoter for genetic engineering of tomato fruit. Hortic Res. 2024. 10.1093/hr/uhae035. 38544552 PMC10967692

[39] Zaidi MA, O’Leary SJB, Gagnon C, Chabot D, Wu S, Hubbard K, et al. A triticale tapetal non-specific lipid transfer protein (nsLTP) is translocated to the pollen cell wall. Plant Cell Rep. 2020;39(9):1185–97. 10.1007/s00299-020-02556-6. 32638075

[40] Fleury C, Gracy J, Gautier MF, Pons JL, Dufayard JF, Labesse G, et al. Comprehensive classification of the plant non-specific lipid transfer protein superfamily towards its sequence–structure–function analysis. Peer J. 2019;7:e7504. 10.7717/peerj.7504. 31428542 PMC6698131

[41] Oppenheimer DG, Herman PL, Sivakumaran S, Esch J, Marks MD. A myb gene required for leaf trichome differentiation in Arabidopsis is expressed in stipules. Cell. 1991;67(3):483–93. 10.1016/0092-8674. 1934056

[42] Marks MD, Feldmann KA. Trichome Development in Arabidopsis thaliana. I. T-DNA tagging of the GLABROUS1 gene. Plant Cell nov. 1989;1(11):1043–50. 10.1105/tpc.1.11.1043. PMC159841 12359885

[43] Ji W, Mandal S, Rezenom YH, McKnight TD. Specialized metabolism by trichome-enriched Rubisco and fatty acid synthase components. Plant Physiol. 2023;191(2):1199–213. 10.1093/plphys/kiac487. 36264116 PMC9922422

[44] Wang Y, Wen J, Liu L, Chen J, Wang C, Li Z. Engineering of tomato type VI glandular trichomes for trans-chrysanthemic acid biosynthesis, the acid moiety of natural pyrethrin insecticides. Metabolic Engineering. 2022;72:188–99. 10.1016/j.ymben.2022.03.007. 35339691

[45] Decaestecker W, Buono RA, Pfeiffer ML, Vangheluwe N, Jourquin J, Karimi M, et al. CRISPR-TSKO: A Technique for Efficient Mutagenesis in Specific Cell Types, Tissues, or Organs in Arabidopsis. The Plant Cell. 2019;31(12):2868–87. 10.1105/tpc.19.00454. 31562216 PMC6925012

[46] Allen F, Crepaldi L, Alsinet C, Strong AJ, Kleshchevnikov V, De Angeli P, et al. Predicting the mutations generated by repair of Cas9-induced double-strand breaks. Nat Biotechnol. 2018(1). 10.1038/nbt.4317. 30480667 PMC6949135

[47] Shen MW, Arbab M, Hsu JY, Worstell D, Culbertson SJ, Krabbe O, et al. Predictable and precise template-free CRISPR editing of pathogenic variants. Nature. 2018;563(7733):646–51. 10.1038/s41586-018-0686-x. 30405244 PMC6517069

[48] Lee JS, Bae SJ, Kim JS, Kim C, Kang BC. A streamlined guide RNA screening system for genome editing in Sorghum bicolor. Plant Methods. 2023;19(1):90. 10.1186/s13007-023-01058-2. 37633915 PMC10463630

[49] Bollier N, Andrade Buono R, Jacobs TB, Nowack MK. Efficient simultaneous mutagenesis of multiple genes in specific plant tissues by multiplex CRISPR. Plant Biotechnol J. 2021;19(4):651–3. 10.1111/pbi.13525. 33305496 PMC8051595

[50] Ali Z, Mahfouz MM, Mansoor S. CRISPR-TSKO: A Tool for Tissue-Specific Genome Editing in Plants. Trends Plant Sci. 2020;25(2):123–6. . 10.1016/j.tplants.2019.12.002 31859038

[51] Nonaka S, Arai C, Takayama M, Matsukura C, Ezura H. Efficient increase of ɣ-aminobutyric acid (GABA) content in tomato fruits by targeted mutagenesis. Sci Rep 1 août. 2017;7(1):7057. 10.1038/s41598-017-06400-y. 28765632 PMC5539196

[52] Ishida K, Yokoyama R. Reconsidering the function of the xyloglucan endotransglucosylase/hydrolase family. Journal of Plant Research. 2022;135(2):145–56. 10.1007/s10265-021-01361-w. 35000024

[53] Wang D, Yeats TH, Uluisik S, Rose JKC, Seymour GB. Fruit softening: revisiting the role of pectin. Trends in Plant Science. 2018;23(4):302–10. 10.1016/j.tplants.2018.01.006. 29429585

[54] Gierlinger N. New insights into plant cell walls by vibrational microspectroscopy. Appl Spectrosc Rev. 2018;53(7):517–51. 10.1080/05704928.2017.1363052. 30057488 PMC6050719

[55] Lescot M, Déhais P, Thijs G, Marchal K, Moreau Y, Van de Peer Y. PlantCARE, a database of plant cis-acting regulatory elements and a portal to tools for in silico analysis of promoter sequences. Nucleic Acids Res. 2002;30(1):325–7. 10.1093/nar/30.1.325. 11752327 PMC99092

[56] Karimi M, Depicker A, Hilson P. Recombinational cloning with plant gateway vectors. Plant Physiol. 2007;145(4):1144–54. 10.1104/pp.107.106989. 18056864 PMC2151728

[57] Musseau C, Jorly J, Gadin S, Sørensen I, Deborde C, Bernillon S, et al. The tomato guanylate-binding protein SlGBP1 enables fruit tissue differentiation by maintaining endopolyploid cells in a non-proliferative state. Plant Cell. 2020;32(10):3188–205. 10.1105/tpc.20.00245. 32753430 PMC7534463

[58] Bres C, Petit J, Reynoud N, Brocard L, Marion D, Lahaye M, et al. The SlSHN2 transcription factor contributes to cuticle formation and epidermal patterning in tomato fruit. Molecular Horticulture. 2022;2(1):14. 10.1186/s43897-022-00035-y. 37789465 PMC10515250

[59] Strati IF, Oreopoulou V. Effect of extraction parameters on the carotenoid recovery from tomato waste. International Journal of Food Science & Technology. 2010;46(1):23. 10.1111/J.1365-2621.2010.02496.X.

[60] Marc M, Lopez C, Viller N, Falourd X, Fanuel M, Marion D, et al. From tomato pomaces biorefinery to biobased shape-memory semicrystalline polyester networks. ACS Sustainable Chemistry & Engineering. 2024;12(6):2191–202. 10.1021/acssuschemeng.3c05713.

